# Public Understanding of and Engagement With Community Health Workers and *Promotores de Salud*: Findings From Two National Surveys

**DOI:** 10.5888/pcd22.240441

**Published:** 2025-07-24

**Authors:** Elizabeth A. Rohan, Julie S. Townsend, Andrea Torres, Hope L. Thompson, Dawn M. Holman, Avid Reza, Felicia Solomon Tharpe, Erin Caple, Ashley Wennerstrom

**Affiliations:** 1 Division of Cancer Prevention and Control, Centers for Disease Control and Prevention, Chamblee, Georgia; 2 Division of Diabetes Translation, Centers for Disease Control and Prevention, Chamblee, Georgia; 3 Center for Community Health Alignment, University of South Carolina Arnold School of Public Health, Columbia, South Carolina; 4 Louisiana State University Health Sciences Center New Orleans, Department of Community Health Science and Policy, School of Public Health and Center for Healthcare Value and Equity, School of Medicine, New Orleans, Louisiana

## Abstract

**Introduction:**

Community health workers (CHWs) can improve health by helping people navigate health care services and systems and connecting them to community services that address nonmedical factors such as food insecurity, housing, and transportation. While CHWs have long been part of the US public health system, there are no data, to our knowledge, on the public’s familiarity and interactions with CHWs.

**Methods:**

We analyzed data from the 2022 Porter Novelli FallStyles and Estilos surveys, online market research surveys of the general public delivered in English (FallStyles) or primarily Spanish (Estilos). Each survey asked the same 4 questions on familiarity and interactions with CHWs, including, for respondents reporting having interacted with CHWs, the types of issues CHWs helped them with.

**Results:**

Of 3,410 FallStyles respondents, 52.1% were familiar with CHWs, and 16.7% had interacted with a CHW. Of 954 Estilos respondents, 62.4% were familiar with CHWs, and 42.0% had interacted with a CHW. Among respondents who indicated being helped by CHWs, the majority encountered CHWs in health care settings (60.8% of FallStyles respondents; 64.2% of Estilos respondents) and less than one-quarter reported encountering CHWs in their community or place of worship (21% of FallStyles respondents; 22.2% of Estilos respondents).

**Conclusion:**

A large proportion of people who are Hispanic or Latino are familiar with or have had interactions with CHWs. The low levels of familiarity with CHWs among FallStyles respondents highlight opportunities for greater public awareness and understanding of the multifaceted role and scope of the CHW workforce across public health, health care, and community settings to enhance the health and wellness of all people.

**Summary**

**What is already known on this topic?**

Community health workers (CHWs) are important members of the public health workforce and can help people prevent and manage chronic diseases. Little is known about the public’s understanding of and interactions with CHWs.

**What is added by this report?**

To our knowledge, this is the first study to use a nationally derived sample to ask people about their knowledge of and interactions with CHWs. Findings may guide development of messages about engagement with CHWs in health care and other settings to enhance access to care, chronic disease management, and preventive services.

**What are the implications for public health practice?**

Public health programs can educate the public on the various roles of CHWs to increase public engagement with CHWs and improve the health and wellness of Americans.

## Introduction

Since the 1960s, community health workers (CHWs) have worked within US public health and community-based organizations to promote health and wellness (1–6). The American Public Health Association defines a CHW as “a frontline public health worker who is a trusted member of and/or has an unusually close understanding of the community served” (7). This trusting relationship facilitates CHWs’ ability to connect people and communities with health and social services. CHWs can also help improve service delivery to people with different backgrounds. CHWs are known by other titles; Hispanic/Latino communities use the term *promotoras (*or *promotores) de salud.*

The Centers for Disease Control and Prevention (CDC) has long recognized the contributions of CHWs in clinical and public health practices. Research has demonstrated that CHWs are effective in increasing cancer screening rates for breast, colorectal, and cervical cancers; helping manage chronic conditions, such as diabetes and cardiovascular disease; and helping manage risk factors, such as high blood pressure and cholesterol (8–12). Hispanic or Latino people in the US have a higher incidence and higher death rates of cervical cancer and liver cancer than the general population (13–15). They also have higher rates of other chronic diseases such as diabetes and hypertension compared with other demographic groups (13,16). CHWs promote health and wellness by addressing nonmedical factors that affect health (6,12,17–21) and are increasingly being engaged to address these challenges in various settings and among various populations (22).

Recognition of CHWs’ contributions to improving health has prompted several large national workforce studies in the last 3 decades (1,23–26). The CHW field now has information about the role of CHWs in the workforce, their demographic characteristics, and their employment characteristics, such as the various agencies where they work (3,5). Also, a recent study of primary care providers found that just over half of providers surveyed had worked with a CHW (27); however, no national studies, to our knowledge, describe the public’s familiarity with CHWs. We used national-level surveys to examine and describe the public’s familiarity and interactions with CHWs, including settings (eg, health care, community) where people encountered CHWs and what services CHWs provided. Understanding how the public interacts with CHWs can help future efforts to educate the public and incorporate CHWs in public health programs.

## Methods

We analyzed data from the 2022 Porter Novelli FallStyles and Estilos surveys, online national market research surveys fielded from September 1 through September 24, 2022 (FallStyles) and September 29 through November 25, 2022 (Estilos) (28). FallStyles was fielded in English to the general public. Estilos was fielded to a respondent panel of people who identified as Hispanic or Latino and was offered primarily in Spanish but also in English, based on respondent preference. The surveys included 4 questions related to CHWs. These questions were initially written in English for FallStyles and then translated into Spanish for Estilos and further reviewed by a native Spanish speaker for accuracy. Data did not include identifiable information. This activity was reviewed by CDC and was conducted consistent with applicable federal law and CDC policy.

### Survey respondents

FallStyles is part of the Porter Novelli ConsumerStyles surveys conducted via the Ipsos Knowledge Panel, an online panel designed to be representative of the noninstitutionalized US population. Recruitment and other methodologic details are available for FallStyles (https://styles.porternovelli.com/consumer-youthstyles) and Estilos (https://styles.porternovelli.com/estilos). FallStyles respondents were recruited from a sample of 4,514 panelists aged 18 years or older who completed the SpringStyles survey. A total of 3,526 adults completed the FallStyles survey, for a participation rate of 78.1%. The unweighted demographic profile of FallStyles respondents was similar to the profile of SpringStyles respondents, with the exception of household size, in which more FallStyles respondents were from households with 2 or fewer people.

Estilos is an annual web-based survey administered by Offerwise through its QueOpinas panel, the largest online panel of Hispanic people in the US, with more than 220,000 active panelists. QueOpinas recruits panels via English-language and Spanish-language network television. Estilos respondents were recruited from a sample of 2,884 panelists aged 18 years or older, and participation was capped at 1,000 participants. 

### Data collection

The CHW-related questions included questions about familiarity with CHWs, settings in which respondents met with CHWs, and activities with which CHWs helped respondents (Appendix). Questions about settings and activities were derived from previous literature defining CHW roles and core competencies (1,5).

#### Variables 

**Familiarity with CHWs.** Respondents were provided a standard definition of a CHW and asked, “Prior to this survey, how familiar were you with what CHWs do?” Response options were on a 5-point scale (from 1 = extremely familiar to 5 = not at all familiar). We grouped respondents who selected responses 1, 2, 3, or 4 into the “familiar” category. Those who selected a response of 5 were categorized as “not familiar.”

**Settings.** Respondents were asked in what settings (if any) CHWs had helped them. Response options were the following: clinic or hospital, social service system, community or place of worship, at home, at a job, no involvement, or “I am a CHW.” Respondents who indicated no involvement with CHWs or identified as CHWs were not asked about receiving CHW services.

**Services.** Respondents who had been helped by a CHW were asked a series of yes/no/don’t know items about how a CHW helped within the health care system (eg, get the medical care I needed, enroll in health insurance) and the types of social services CHWs helped people obtain (eg, housing/shelter, food, employment).

### Data analysis

For both surveys, we calculated descriptive statistics overall and by level of familiarity with CHWs and interactions with CHWs, comparing respondents by demographic characteristics. We used χ^2^ tests for statistical comparisons. We analyzed each survey separately, given differences in recruitment strategies and panel designs; however, we tabulated findings from both surveys together for nonstatistical comparisons. We used SAS 9.4 (SAS Institute, Inc) to conduct analyses. *P* values ≤.05 were considered significant. We incorporated into both analyses survey weights designed to weight the data to match proportions of either the US Current Population Survey or the American Community Survey. We used SAS survey procedures for analysis, producing weighted counts and percentages. We excluded from the analysis 43 FallStyles and 45 Estilos respondents (unweighted count) who reported being CHWs themselves. Fifty-three FallStyles respondents did not respond to the question about being helped by a CHW, leaving an unweighted count of 3,430 respondents remaining in the analysis. All results are reported as weighted counts.

## Results

Among FallStyles respondents, 65.4% identified as White, 13.0% as Hispanic or Latino, and 11.8% as Black (Table 1). Nearly one-third (31.8%) were aged 60 years or older, and 26.4% were aged 30 to 44 years. Fifty-one percent of respondents were female, 35.7% had at least a college diploma, 28.2% had only a high school diploma, and 45.9% reported a household income less than $50,000. Among Estilos respondents, a little more than one-third (34.4%) were aged 30 to 44 years, and nearly one-quarter were aged 45 to 59 years (24.6%). Half (50.9%) of Estilos respondents were female, 43% had a household income below $50,000, 27.1% had less than a high school diploma, and 27.4% had only a high school diploma.

**Table 1:** Demographic Characteristics of FallStyles and Estilos Respondents, 2022

Characteristic	FallStyles, no. (%)^a^^,^ b	Estilos, no. (%)^a^^,^ c
**Total**	3,410	954
**Race and ethnicity^d^**		
American Indian or Alaska Native	46 (1.3)	—e
Asian	206 (6.1)	
Black or African American	400 (11.8)	
Hispanic or Latino	442 (13.0)	
Native Hawaiian or Pacific Islander	11 (0.3)	
White	2,224 (65.4)	
I identify equally with more than one	72 (2.1)	
**Age, y**		
18–29	660 (19.3)	219 (22.9)
30–44	899 (26.4)	329 (34.4)
45–59	768 (22.5)	235 (24.6)
^3^60	1,083 (31.8)	172 (18.0)
**Sex**		
Male	1,657 (48.6)	469 (49.1)
Female	1,741 (51.1)	486 (50.9)
**Annual household income, $^f^**		
<25,000	425 (12.5)	167 (17.5)
25,000–49,999	1,138 (33.4)	242 (25.4)
50,000–74,999	1,064 (31.2)	284 (29.8)
^3^75,000	783 (23.0)	261 (27.4)
**Education**		
Less than high school diploma	308 (9.0)	259 (27.1)
High school graduate	961 (28.2)	261 (27.4)
Some college	923 (27.1)	242 (25.4)
College diploma or more	1,218 (35.7)	192 (20.1)
**Region**		
Northeast	582 (17.1)	132 (13.9)
South	1,303 (38.2)	364 (38.2)
Midwest	710 (20.8)	92 (9.7)
West	815 (23.9)	365 (38.2)

a Counts and associated percentages were weighted by using weights developed to match demographic characteristics found in the US Current Population Survey or American Community Survey. Due to rounding, some weighted counts may not add to the total weighted count. No statistical comparisons across surveys were performed due to methodologic differences in survey design.

b Forty-three FallStyles who reported being community health workers themselves were excluded. Additionally, 53 FallStyles respondents (unweighted count) were excluded because they did not respond to the question about interaction with community health workers.

c Forty-five Estilos respondents (unweighted count) who reported being community health workers themselves were excluded.

d The question on race and ethnicity asked, “Which race or ethnicity do you most identify with [select one]?” Nine (unweighted count) FallStyles respondents did not provide their race and ethnicity (weighted count = 20).

e Estilos recruitment was based on people who self-identified as Hispanic or Latino.

f Household income was coded in Estilos to the nearest tens of thousands and further combined for this analysis. In Estilos, the largest category was $80,000.

Of FallStyles respondents, 52.1% were familiar with CHWs, and 16.7% reported having interacted with a CHW (Table 2). By age, rates of familiarity with CHWs were highest among FallStyles respondents aged 60 years or older and lowest among respondents aged 18 to 29 years (54.1% vs 45.0%; *P* = .01). FallStyles respondents reporting household income levels of $75,000 or more had greater familiarity with CHWs compared with respondents reporting an annual income of less than $25,000 (56.2% vs 47.9%; *P* = .02). Conversely, FallStyles respondents in the lowest income category, compared with respondents in the highest income category, more frequently reported interactions with CHWs (24.6% vs 12.6%; *P* < .001). Familiarity with CHWs was greater among FallStyles respondents reporting a college diploma or more than among those reporting less than a high school diploma (60.6% vs 39.6%; *P* < .001).

**Table 2:** Selected Demographic Characteristics of FallStyles and Estilos Respondents by Familiarity With CHWs and Whether They Interacted With CHWs, 2022

Characteristic	Familiar with CHWs				Interacted with CHWs			
	FallStyles		Estilos		FallStyles		Estilos	
	No. (%)^a^^,^ b	*P* value^c^	No. (%)^a^^,^ d	*P* value^c^	No. (%)^a^^,^ b	*P* value^c^	No. (%)^a^^,^ d	*P* value^c^
**All**	1,771 (52.1)	—	595 (62.4)	—	570 (16.7)	—	401 (42.0)	—
**Age, y**								
18–29	297 (45.0)	.01	117 (53.5)	.23	109 (16.5)	.91	84 (38.3)	.35
30–44	481 (53.9)		234 (71.3)		144 (16.0)		168 (51.0)	
45–59	407 (53.3)		147 (62.6)		128 (16.7)		89 (37.7)	
≥60	586 (54.1)		97 (56.3)		189 (17.5)		60 (35.1)	
**Annual household income, $^e^**								
<25,000	203 (47.9)	.02	93 (55.9)	.48	105 (24.6)	<.001	63 (37.9)	.14
25,000–49,999	554 (48.9)		148 (61.0)		183 (16.1)		96 (39.8)	
50,000–74,999	575 (54.2)		171 (60.4)		184 (17.3)		101 (35.6)	
≥75,000	439 (56.2)		183 (70.1)		98 (12.6)		140 (53.5)	
**Education**								
Less than high school diploma	122 (39.6)	<.001	162 (62.6)	.48	49 (15.9)	.21	128 (49.4)	.05
High school graduate	408 (42.8)		144 (54.9)		143 (14.8)		81 (30.9)	
Some college	506 (54.8)		155 (64.1)		178 (19.2)		89 (36.6)	
College diploma or more	736 (60.6)		135 (70.0)		201 (16.5)		103 (53.7)	
**Region**								
Northeast	285 (49.2)	.33	61 (46.0)	.01	88 (15.2)	.15	45 (34.2)	.43
South	668 (51.4)		201 (55.1)		243 (18.7)		139 (38.2)	
Midwest	388 (54.8)		66 (71.2)		119 (16.8)		40 (43.7)	
West	430 (53.0)		268 (73.4)		120 (14.7)		176 (48.2)	

Abbreviations: — , does not apply; CHW, community health worker.

a Counts and associated percentages were weighted by using weights developed to match demographic characteristics found in the US Current Population Survey or American Community Survey. Due to rounding, some weighted counts may not add to the total weighted count. No statistical comparisons across surveys were performed due to methodologic differences in survey design.

b Nine (unweighted count) FallStyles respondents did not answer the question about familiarity with CHWs; 43 FallStyles (unweighted count) who reported being community health workers themselves were excluded. Additionally, 53 FallStyles respondents (unweighted count) were excluded because they did not respond to the question about involvement with community health workers.

c Determined by χ^2^ test; *P* values ≤.05 were considered significant.

d 45 Estilos respondents (unweighted counts) who reported being community health workers themselves were excluded.

e Annual household income was coded in Estilos to the nearest tens of thousands and further combined for this analysis. In Estilos, the largest category was ≥$80,000.

Of Estilos respondents, 62.4% were familiar with CHWs, and 42.0% reported having interacted with a CHW. The percentage of Estilos respondents who interacted with CHWs was higher among respondents with a college diploma or more than those with less than a high school diploma (53.7% vs 49.4%, *P* = .05). The only other significant difference in demographic characteristics among Estilos respondents was that those residing in the West and Midwest reported more familiarity with CHWs than respondents in other regions (range across all regions, 46.0%–73.4%; *P* = .01).

### Settings in which respondents had interactions with CHWs

**Figure 1:**
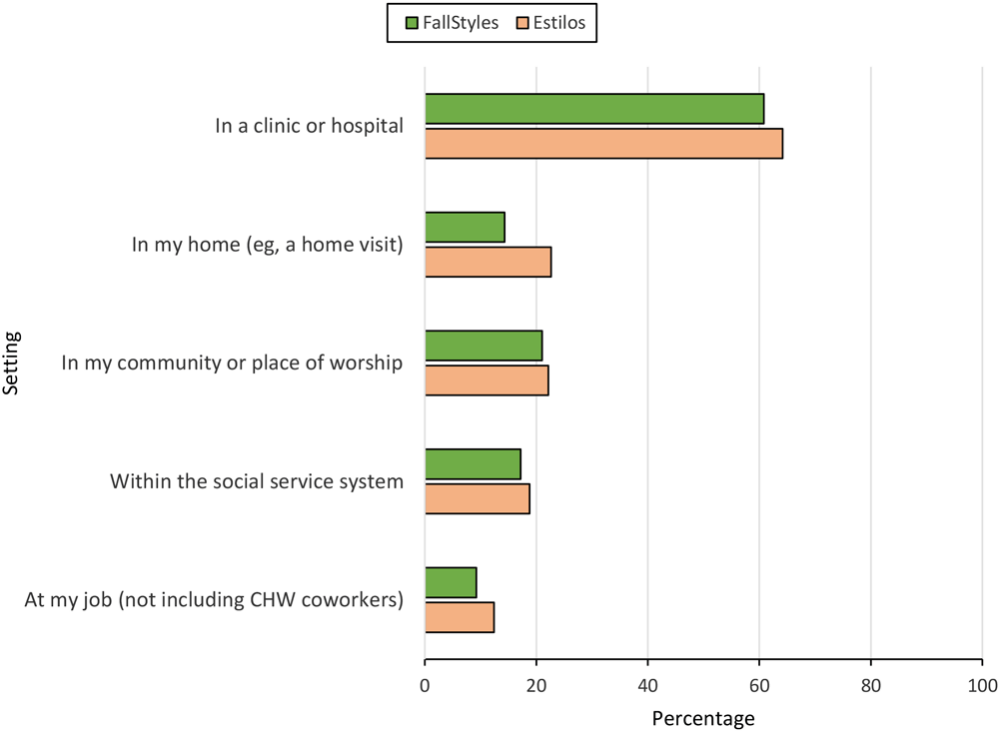
Settings in which community health workers were encountered, US, 2022. Data were obtained from respondents in 2 national surveys: FallStyles, conducted in English in September 2022 among the general public, and Estilos, conducted among a panel of people who identified as Hispanic or Latino and offered primarily in Spanish, in September through November 2022. Data are from a subset of respondents who indicated they had been helped by a community health worker (FallStyles, n = 570; Estilos n = 401).

The majority of both FallStyles and Estilos respondents who indicated they had been helped by a CHW reported that their interactions occurred in health care settings (60.8% and 64.2%, respectively) (Figure 1). Less than a quarter of respondents who had been helped by a CHW reported interactions in their community or place of worship (21% FallStyles; 22.2% Estilos).

**Table:** 

Settings in which CHWs were encountered	FallStyles, %	Estilos, %
At a clinic or hospital	60.8	64.2
In my home (eg, a home visit)	14.3	22.7
In my community or place of worship	21.0	22.2
Within the social service system	17.2	18.8
At my job (not including CHW coworkers)	9.3	12.4

### Activities in which CHWs assisted respondents

**Figure 2:**
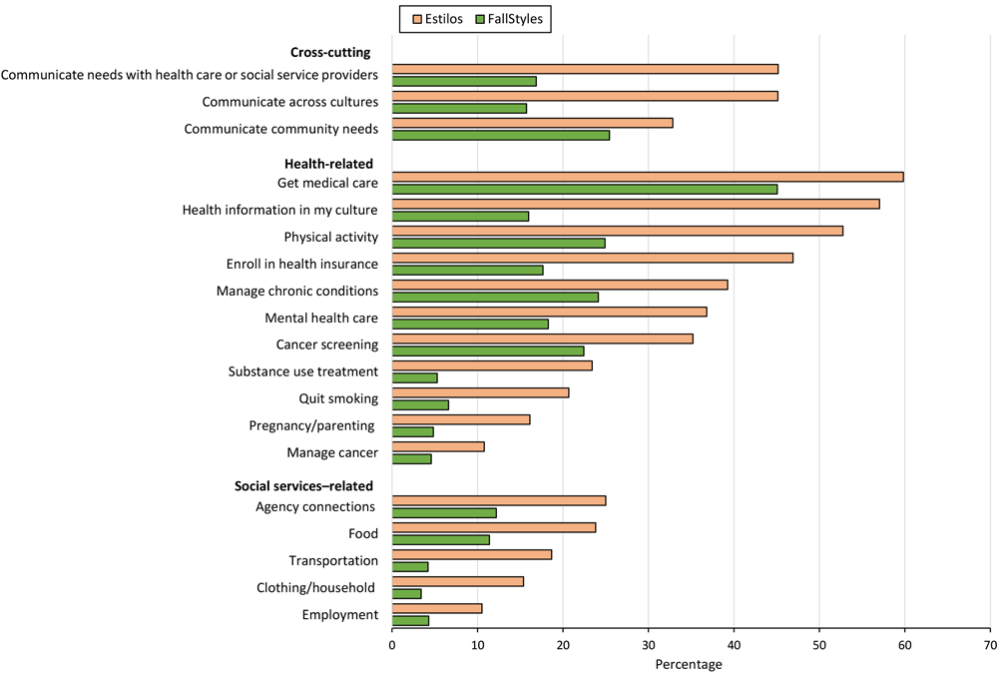
Activities in which community health workers assisted survey respondents, US, 2022. Data were obtained from 2 national surveys: FallStyles, conducted in English in September 2022 among the general public and Estilos, conducted among a panel of people who identified as Hispanic or Latino in September through November 2022. Data are from a subset of respondents who indicated they had been helped by a community health worker. For the question on social services, FallStyles n = 565 and Estilos n = 401. For the question on health-related and cross-cutting activities, FallStyles n = 570, and Estilos n = 401. For social services–related items, only items for which at least 10% of either group (FallStyles or Estilos) indicated being helped with are shown.

Among survey respondents indicating CHW involvement, respondents from both surveys reported that CHWs helped them with various activities across health-related and social service–related issues; however, some activities were cross-cutting. For example, 15.7% of FallStyles respondents reported that CHWs helped them communicate across cultures to better access health and social services, 16.9% reported CHWs helped them communicate their needs with health care or social service providers, and 25.4% reported CHWs helped them communicate their community’s needs (Figure 2). Nearly half (45.1%) of Estilos respondents that reported CHWs helped them communicate across cultures to better access health and social services and communicate needs with health care or social service providers. One-third (32.9%) of Estilos respondents reported CHWs helped them communicate their community’s needs.

**Table:** 

Activity	FallStyles, %	Estilos, %
**Cross-cutting activities**		
Communicate needs with health care or social service providers	16.9	45.2
Communicate across cultures	15.7	45.1
Communicate community needs	25.4	32.9
**Health-related activities**		
Get medical care	45.1	59.8
Health information in my culture	16.0	57.0
Physical activity	24.9	52.7
Enroll in health insurance	17.7	46.9
Manage chronic conditions	24.1	39.3
Mental health care	18.3	36.8
Cancer screening	22.4	35.2
Substance use treatment	5.3	23.4
Quit smoking	6.6	20.7
Pregnancy/parenting	4.8	16.1
Manage cancer	4.6	10.8
**Social service–related activities**		
Agency connections	12.2	25.0
Food	11.4	23.8
Transportation	4.2	18.7
Clothing/household	3.4	15.4
Employment	4.3	10.5

FallStyles respondents indicating they had been helped by CHWs reported that CHWs helped them obtain medical care (45.1%), increase physical activity (24.9%), and manage chronic health conditions (24.1%) (Figure 2). Fewer FallStyles respondents reported that CHWs helped them quit smoking (6.6%), obtain substance use treatment (5.3%), obtain home visits during pregnancy and/or help with parenting (4.8%), or manage cancer (4.6%). Most Estilos respondents indicating they had been helped by CHWs reported that CHWs helped them obtain medical care (59.8%), obtain linguistically and culturally concordant health information (57.0%), increase physical activity (52.7%), and enroll in health insurance (46.9%). Fewer Estilos respondents reported receiving assistance from CHWs to obtain home visits during pregnancy or help with parenting (16.1%) or manage cancer (10.8%).

FallStyles respondents indicating they had been helped by a CHW reported that CHWs helped them obtain connections with agencies (12.2%), food (11.4%), and elder care (7.5%) (Figure 2). Estilos respondents indicating they had been helped by a CHW reported that CHWs helped them obtain connections with agencies (25.0%), food (23.8%), and transportation (18.7%).

## Discussion

We analyzed data from 2 nationally derived surveys to conduct the first, to our knowledge, large-scale assessment of familiarity with CHWs among the general public (FallStyles) and the Hispanic or Latino population (Estilos). Overall, we found that FallStyles respondents with higher incomes and greater education were more familiar with CHWs, while those with lower incomes more often reported interacting with CHWs.

Our study has several implications. Although the surveys are not directly comparable, the finding that a higher percentage of Estilos respondents than FallStyles reported being familiar with and having received support from a CHW suggest that CHWs have established inroads into reaching Hispanic or Latino populations. The finding that Estilos respondents interacted with CHWs at higher rates than FallStyles respondents across all response categories may indicate that Hispanic or Latino populations need more support to navigate multiple complex health and social needs and may lack trust in the health care system (29).

Overall, our findings suggest that public health programs focused on preventing chronic diseases may consider engaging CHWs to provide outreach and education. For example, CHWs may be helpful in promoting cancer screening among Hispanic or Latino populations, since Hispanic adults have been found to be less likely than non-Hispanic adults to be up-to-date with cervical and colorectal cancer screening (30). CHWs have long been part of outreach and recruitment efforts in CDC’s cancer prevention and control programs as an evidence-based strategy (8,9) to help individuals across the cancer continuum, from screening to treatment referral to long-term survivorship.

Our findings align with recent studies of the CHW workforce that pointed to a need for greater public awareness and understanding of CHWs’ multifaceted roles, scope, and locations of employment (26,31). Although FallStyles and Estilos respondents most frequently identified receiving CHW support in health care settings, a recent survey of US-based CHWs found that most CHW work took place in community-based settings, rather than clinical settings (26). Further studies could help explain this discrepancy. Even when individuals are familiar with and benefit from community-level public health activities, they may not realize that a CHW, as opposed to another professional, performed the work. Greater public awareness of CHWs’ community-level roles could help ensure that individuals seek CHW support for social and community needs, rather than strictly medical needs.

Additionally, education for health providers about CHWs’ roles could support improved interprofessional collaboration and health care team integration. A recent survey of primary care providers revealed that those who had not previously worked with CHWs believed having additional information about CHW roles and functions would be helpful (27). Even providers who were familiar with CHWs believed lack of information about how CHWs can collaborate with other health professionals was a barrier to team integration (27). Ensuring that health care providers understand CHWs’ scope of practice, the extent of their professional training, and their unique skills may promote uptake of CHW services and alleviate common misconceptions about CHWs’ activities.

### Strengths and limitations

This study has several strengths. To our knowledge, this is the first study using nationally derived samples of individuals to understand their familiarity and interactions with CHWs. The Estilos survey was administered to Hispanic/Latino adults, with the option of responding in either Spanish or English, allowing for an examination of a population with health disparities across several chronic conditions. In addition, the FallStyles survey had a high participation rate and a relatively large sample size.

This study has several limitations. First, self-reported data can be subject to recall bias and social desirability bias. Second, we could not capture all possible places that an individual can encounter a CHW (eg, health departments) because free text is not a feature of Porter Novelli surveys. Third, although FallStyles and Estilos surveys are weighted to match the US Census Bureau’s proportions, people who agree to participate in online survey panels could be systematically different from others, so our findings may not be generalizable given that population-based probability sampling methods were not used. Finally, because of differences in survey methodology and population sampled between FallStyles and Estilos, we could not perform statistical tests to assess differences between the 2 groups of survey respondents. Despite these limitations, this study contributes to our understanding of the degree to which the general public and Hispanic or Latino people are familiar with and how they interact with CHWs.

### Conclusion

A strategic focus on including CHWs in public health awareness campaigns, coupled with the reach of public health programs already engaging CHWs, could enhance the public’s knowledge of the CHW workforce and ultimately increase public engagement with CHWs.

## Appendix Questions About Community Health Workers on FallStyles Survey and Estilos Survey, 2022

**Table:**

Question no.	Question	Response options
Question 1	Community health workers (CHWs) are frontline public health workers who are trusted members of the community or have a very good understanding of the community they serve. They help people get the health and social services they need. CHWs understand cultural beliefs, behaviors, and needs of their clients to better connect the community and the healthcare system. CHWs may be referred to by many titles. For this survey, please think about the role described here. Prior to this survey, how familiar were you with what CHWs do?	Select only one: Extremely familiar Moderately familiar Somewhat familiar Slightly familiar Not at all familiar
Question 2	In what settings have you been helped by a CHW?	Select all that apply: Within a clinic or hospital Within the social service system In my community or place of worship In my home (eg, a home visit) At my job (not including CHW coworkers) No involvement with CHWs [if this was selected, no other options could be selected and survey ended] I am a CHW [if this was selected, no other options could be selected and survey ended]
Question 3	CHWs have helped me:	
	3.1. Communicate across cultures to help me better connect with health care or social services	Select one [for each question]: Yes No Don’t know
	3.2. Get health information in my primary language and that reflects my culture.	
	3.3. Get the medical care I needed.	
	3.4. Get the mental health care I needed.	
	3.5. Get treatment for my substance abuse or addiction.	
	3.6. Get a screening for cancer, such as a mammogram or colonoscopy.	
	3.7. Enroll in health insurance.	
	3.8. Speak up for my needs with health care providers or social service providers.	
	3.9. Speak up for the needs of my community.	
	3.10. Quit smoking or help me find services to help me quit.	
	3.11. Manage my cancer.	
	3.12. Manage my other chronic conditions (eg, arthritis, diabetes, high blood pressure, chronic obstructive pulmonary disease).	
	3.13. Increase my physical activity level or eat healthier foods.	
	3.14. Get home visits to support my pregnancy and/or help me with parenting my infant or young child.	
Question 4	What types of social services have CHWs helped you with?	Select all that apply: Housing/shelter (including energy bills) Food (including food banks, SNAP, WIC) Employment and income Clothing and/or household items Childcare and/or parenting Elder care A legal issue Connected me to another agency for help Transportation Other service Didn’t help me with social services [if this was selected, no other options could be selected]

Abbreviations: SNAP, Supplemental Nutrition Assistance Program; WIC, Special Supplemental Nutrition Program for Women, Infants, and Children.
